# Magnetic resonance imaging features of invasive breast cancer association with the tumour stromal ratio

**DOI:** 10.1371/journal.pone.0290772

**Published:** 2023-08-25

**Authors:** Nazimah Ab Mumin, Marlina Tanty Ramli Hamid, Shamsiah Abdul Hamid, Seow-Fan Chiew, Mohd Shahril Ahmad Saman, Kartini Rahmat

**Affiliations:** 1 Faculty of Medicine, Department of Radiology, Universiti Teknologi MARA, Selangor, Malaysia; 2 Faculty of Medicine, Department of Biomedical Imaging, University of Malaya, Kuala Lumpur, Malaysia; 3 Faculty of Medicine, Department of Pathology, University of Malaya, Kuala Lumpur, Malaysia; 4 Faculty of Medicine, Department of Public Health, Universiti Teknologi MARA, Selangor, Malaysia; Affiliated Hospital of Nanjing University of Chinese Medicine: Jiangsu Province Academy of Traditional Chinese Medicine, CHINA

## Abstract

**Objective:**

To assess the association between breast cancer tumour stroma and magnetic resonance imaging (MRI) features.

**Materials and methods:**

A total of 84 patients with treatment-naïve invasive breast cancer were enrolled into this retrospective study. The tumour stroma ratio (TSR) was estimated from the amount of tumour stroma in the pathology specimen of the breast tumour. The MRI images of the patients were analysed based on Breast Imaging Reporting and Data Systems (ACR-BIRADS) for qualitative features which include T2- weighted, diffusion-weighted images (DWI) and dynamic contrast-enhanced (DCE) for kinetic features. The mean signal intensity (SI) of Short Tau Inversion Recovery (STIR), with the ratio of STIR of the lesion and pectoralis muscle (L/M ratio) and apparent diffusion coefficient (ADC) value, were measured for the quantitative features. Correlation tests were performed to assess the relationship between TSR and MRI features.

**Results:**

There was a significant correlation between the margin of mass, enhancement pattern, and STIR signal intensity of breast cancer and TSR. There were 54.76% (n = 46) in the low stromal group and 45.24% (n = 38) in the high stromal group. A significant association were seen between the margin of the mass and TSR (p = 0.034) between the L/M ratio (p <0.001), and between STIR SI of the lesion and TSR (p<0.001). The median L/M ratio was significantly higher in the high TSR group as compared to the lower TSR group (p < 0.001).

**Conclusion:**

Breast cancer with high stroma had spiculated margins, lower STIR signal intensity, and a heterogeneous pattern of enhancement. Hence, in this preliminary study, certain MRI features showed a potential to predict TSR.

## Introduction

Breast cancer is the world’s number one cause of cancer death [1]. Understanding breast cancer behaviour is important for personalized medicine, tailored therapy, prediction of survival and reduction of mortality. Currently, magnetic resonance imaging (MRI) is the most sensitive imaging study to detect, stage, and characterize breast cancer [2].

Many factors contribute to breast cancer’s aggressiveness, including tumour subtypes, angiogenesis, hormone receptor status, tumour metabolism and cell proliferation [3]. Aside from the molecular markers, breast tumour fibrosis has been a focus in breast cancer pathology studies [4,5]. Heterogeneity within a single tumour is a major cause of therapy resistance and treatment failure [3]. Invasive breast cancer is divided into four main subtypes (Luminal A, Luminal B, HER-2 enriched, and triple-negative), according to different genetic expressions [6–8]. However, within the same subtype, patients were noted to have different prognoses and survival [9,10].

Cancer cell-associated stroma, namely fibroblasts and extracellular matrix influences tumour cell invasion, metastasis and malignant behaviour. Cancer-associated fibroblast (CAFs) which are intrinsic within the tumour cell stroma increases the tumour stromal component. Remodeling of the extracellular matrix and disruption of epithelial tissue were secondary to the increased stromal component secreted by cancer cells [4,5]. Tumour stromal ratio (TSR) has been proven to be an independent prognostic factor in determining relapse-free periods in invasive breast cancer, especially in triple-negative cancers [4].

Pathological studies have reported a positive correlation between high tumour stromal content to poor prognosis of invasive breast cancer, especially in triple-negative cancers [4,5,11]. However, for ER-positive breast cancers, improved outcomes were associated with a high tumour stroma [12]. In these studies, the histopathology samples were obtained via core needle biopsy or vacuum-assisted biopsy and were only able to capture a small part of a larger and heterogeneous tumour.

MRI breast can portray the whole tumour as signal intensities and the image data are of the tumour in its entirety. There were only few publications in MRI and breast cancer TSR [13,14]. MRI breast which can potentially capture phenotypical features of the cancer in its entirety may be a useful adjunctive tool to classify TSR and consequently predict tumour behavior. Previous studies have suggested that Short Tau Inversion Recovery (STIR) and dynamic contrast enhanced (DCE) images reflect the tumour stromal component of breast cancer [13–15].

Breast cancer, until now remains a challenge in cancer treatment, caused by intratumoural, as well as spatial and temporal heterogeneity. By deciphering one of the important components, namely tumour stroma, through identification of its distinctive MRI features, we can better understand breast tumour complexity. Hence, in this study, we aimed to investigate the MRI features that are associated with the tumour stroma.

## Methodology

This is a retrospective study of female patients with histopathological-proven treatment-naive invasive breast carcinoma who underwent MRI breast from January 2018–December 2020 in University Malaya Medical Centre (UMMC). We excluded cases that underwent neoadjuvant chemotherapy, incomplete pathological data, other type of invasive breast cancer aside from non-special type, and cases with technical error.

The sample size was determined based on Yamaguichi *et al* [15] for TSR and Roganovic *et al* [16] for MRI in detecting breast carcinoma, noted that the sensitivity were 66.67% and 93.1% respectively. Alpha was set at 0.05 and power was at 80%. Two proportion formula via Power Sample Size calculator was used and minimum sample number of 35 patients per arm needed. After considering 20% of attrition rate, total of 84 patients were included for both arms.

All cases were anonymized.

### Pathological study

In this study, the TSR was based on the estimation of the amount of tumour stroma in the primary untreated breast tumour. The cases were from resection and biopsy samples. The included cases with routine Haematoxylin and Eosin (H + E) stained slides were analysed by a pathologist with 5 years of experience. Scoring of TSR were performed using conventional microscope by direct visualization or eyeballing of tumour in routine H+E stained slides. First, a 4 × objective (field diameter 0.1 mm) was used to select the most stroma abundant area within the tumour in the whole tissue slide. The 10 × objective (field diameter r0.25) was used subsequently to assess the percentage of stroma. To ensure only tumour stroma and not supportive stroma was analysed, only microscopic field of vision with the cancer cells that present on all four sides of images were selected. As the breast cancers were known to be heterogeneous, the proportion of stromal areas in the tumour are often variable. Only the tissue section with the highest amount of stroma were selected (7, 18).

The TSR was classified into high stroma group (>50% stromal tissue) or low stromal group (<50% stromal tissue). Areas of necrosis, mucin deposition, and in-situ components were excluded from analysis.

### Radiological study

MRI scan performed using 3.0Tesla Signa^®^ HDx MR Systems (General Electrics (GE) Healthcare) in 31.0% (n = 26) of cases or a 3.0Tesla MAGNETOM Prisma A Tim + Dot System (Siemens Healthcare) in 69.0% (n = 58) of cases, with a dedicated two-channel breast coil with intravenous gadolinium 10cc at 2ml/sec. The imaging parameters for MRI scanners is provided in Supplementary Material. Three radiologists (3–8 years’ experience) analysed the images in consensus which includes interpretation of the T2W, STIR, apparent diffusion coefficient (ADC) (b = 0 and 800), and DCE sequences. The images were analysed based on Breast Imaging Reporting and Data Systems (BI-RADS) [17].

One of the readers (NAM) manually traced the region of interest (ROI) of the invasive breast cancer on STIR and ADC images. The mean signal intensity (SI) and ADC value of each lesion were measured. ROI placement were made over the most enhancing part of the lesion. The SI of the lesion to the pectoralis muscle ratio (L/M ratio) were measured. ROI size standardisation of 3 x 3 mm were used. In addition, the kinetic parameters which was automatically derived from the Syngo software (initial phase: fast, medium, slow and delayed phase: wash-out, plateau, persistent) were collected. The interpretation of the slope of kinetic curve was based on ACR-BIRADS atlas [17].

The authors had no access to the information that could identify individual participants during or after data collection.

### Statistical analysis

Data were collected onto an excel spreadsheet. Statistical analyses were performed using SPSS version 25. Descriptive analyses were performed. The association using chi-square test were made between tumour stroma (between high- or low-stroma group) and the tumour receptor status, histological grade, intrinsic subtype, MRI findings of T2 signal intensity, and mass features. Correlation were tested with Kendall’s tau-b correlation test. Wilcoxon-Signed rank test was performed to determine significant difference between high and low tumour stroma groups in STIR signal intensity (SI), STIR of lesion to pectoralis muscle ratio (L/M ratio), kinetic curve pattern and ADC value.

In all tests, *p*-values < 0.05 was considered significant.

### Ethics approval

Ethics approval was obtained from the UMMC medical ethics committee (ID 2021426–10090).

Informed consent was waived in view of retrospective nature of the study.

## Results

There were a total of 84 cases of invasive breast carcinoma (all non-special type (NST)) included, with mean age of 53.9 (26–78). There were 54.76% (n = 46) in the low stromal group and 45.24% (n = 38) in the high stromal group. The demography and tumour characteristics were tabulated in Table 1.

**Table 1:** Demography and tumour characteristics of the study population.

**Demography**	**Mean (SD)**
Age	53.9 (11.78)
Tumour size (cm)	2.46 (1.21)
**Tumour Grade (Blooms Richardson)**	**N (%)**
Grade 1	9 (10.7%)
Grade 2	49 (58.3%)
Grade 3	25 (29.8%)
**Receptor status**	**N (%)**
Oestrogen-receptor positive	65 (77.4%)
Oestrogen-receptor negative	18 (21.4%)
Progesterone- receptor positive	62 (73.8%)
Progesterone- receptor positive	21 (25.0%)
HER-2 positive	27 (32.1%)
HER-2 negative	55 (65.5%)
**Tumour subtype**	
Luminal A	47 (56.0%)
Luminal B	20 (23.8%)
HER-2 enriched	8 (9.5%)
Triple negative	9 (10.7%)

### MRI qualitative features of the breast cancer mass

The majority of the MRI features were of suspicious characteristics, which were irregular in shape (76.2%; n = 64), spiculated or irregular margin (59.5%; n = 50, and 32.1%, n = 27 respectively), and heterogeneous enhancement (66.7%; n = 56). The majority of kinetics slope assessment were fast in contrast uptake (42.9%; n = 36) and plateau or wash out in the delayed phases (48.8%; n = 41, and 39.3%; n = 33). T2W SI were mostly low or isointense (47.6% and 45.2%). Table 2 is showing the qualitative MRI features in the low and high TSR groups.

**Table 2:** Qualitative MRI features of the mass in the low and high TSR groups.

T2W SI	N (%)	Low TSR	High TSR	p-value
Low intensity	40 (47.6%)	20	20	0.309
Iso intensity	38 (45.2%)	21	17	
High intensity	6 (7.1%)	5	1	
**Shape**				0.215
Oval	6 (7.1%)	5	1	
Round	14 (16.7%)	9	5	
Irregular	64 (76.2%)	32	32	
**Margin**				**0.027** *
Circumscribed	7 (8.3%)	6	1	
Irregular	27 (32.1%)	17	10	
Spiculated	50 (59.5%)	23	27	
**Enhancement pattern**				**0.019** *
Homogeneous	12 (14.3%)	11	1	
Heterogeneous	56 (66.7%)	25	31	
Rim enhancement	15 (17.9%)	9	6	
Dark internal septation	1 (1.2%)	1	0	
**Initial phase (kinetic curve)**				0.286
Slow	21 (25.0%)	9	12	
Medium	27 (32.1%)	14	13	
Fast	36 (42.9%)	23	13	
**Delayed phase (kinetic curve)**				0.139
Persistant	10 (11.9%)	6	4	
Plateau	41 (48.8%)	18	23	
Washout	33 (39.3%)	22	11	

*p-value <0.05.

A medium positive association were seen between margin of the mass and TSR with τb = 0.226, p = 0.034. No correlation between TSR and mass shape (p = 0.100) or enhancement (p = 0.269).

## MRI quantitative features

Table 3 is showing the MRI quantitative values of low and high TSR and each scanner.

**Table 3:** MRI quantitative features in each MRI scanner and low and high TSR groups.

	**GE (n = 26)** **Median (IQR)**	**Low TSR (n = 18)** **Median (IQR)**	**High TSR (n = 8)** **Median (IQR)**
**STIR lesion**	849.00 (540.5)	875.50 (499.3)	689.00 (457.0)
**L/M ratio**	4.00 (1.86)	4.50 (2.49)	3.92 (1.43)
**ADC value (x10-3 mm2/s)**	0.72 (0.29)	0.70 (0.25)	0.90 (0.40)
	**Siemens (n = 58)** **Median (IQR)**	**Low TSR (n = 28)** **Median (IQR)**	**High TSR (n = 30)** **Median (IQR)**
**STIR lesion**	174.00 (94.5)	195.00 (102.0)	129.50 (71.5)
**L/M ratio**	4.78 (2.77)	5.54 (3.67)	5.00 (1.85)
**ADC value (x10-3 mm2/s)**	0.80 (0.25)	0.80 (0.20)	0.85 (0.33)

There was a statistically significant negative correlation between L/M ratio and TSR with τb = -0.438, p <0.001. Significant negative correlation also seen between STIR SI of lesion and TSR τb = -0.367, p<0.001.

The median in L/M ratio were significantly higher in the high TSR group as compared to the lower TSR group in both scanners (GE and Siemens) (z = -7.569, p < 0.001). Median TSR was 4.5 (IQR = 2.49) and 3.922(IQR = 1.43), respectively, for GE. Similar results with Siemens, 5.54 (IQR = 3.67) and 5.00(IQR = 1.85), respectively.

No significant correlation between TSR and ADC value.

### Other correlation

A significant medium positive correlation was seen between HER-2 positive and high TSR with τb = 0.245, p = 0.026.

Table 4 is showing the distribution of receptor status in each TSR group.

**Table 4:** TSR groups and tumour receptor status.

Receptor status	Low TSR (%,n)	High TSR(%,n)	p-value
ER-positive	51.5% (34)	48.5% (32)	0.255
ER-negative	66.7% (12)	33.3% (6)	
PR-positive	52.4% (33)	47.6% (30)	0.450
PR-negative	38.1% (8)	61.9% (13)	
HER2- positive	37.0% (10)	73.0% (17)	**0.026** *
HER2- negative	63.2% (36)	36.8% (21)	

*p value <0.05.

## Discussion

In this study, we investigated the association between tumour stroma and MRI breast features. We found that there is a correlation between margin of mass, enhancement pattern, and STIR signal intensity of the breast cancer and TSR. TSR has been shown to be a prognostic parameter in breast cancer patients, independent of the clinicopathological parameters and therapy [4]. Pathological research groups has divulged in the tumour microenvironment subject, including tumour stroma and fibrosis, with more evidence to suggest that tumour microenvironment influences malignant cancer behaviour, which includes its progression, invasion and metastasis [18]. Low TSR is associated with poor prognosis in breast cancer for hormone positive cancers [19].

**Fig 1:**
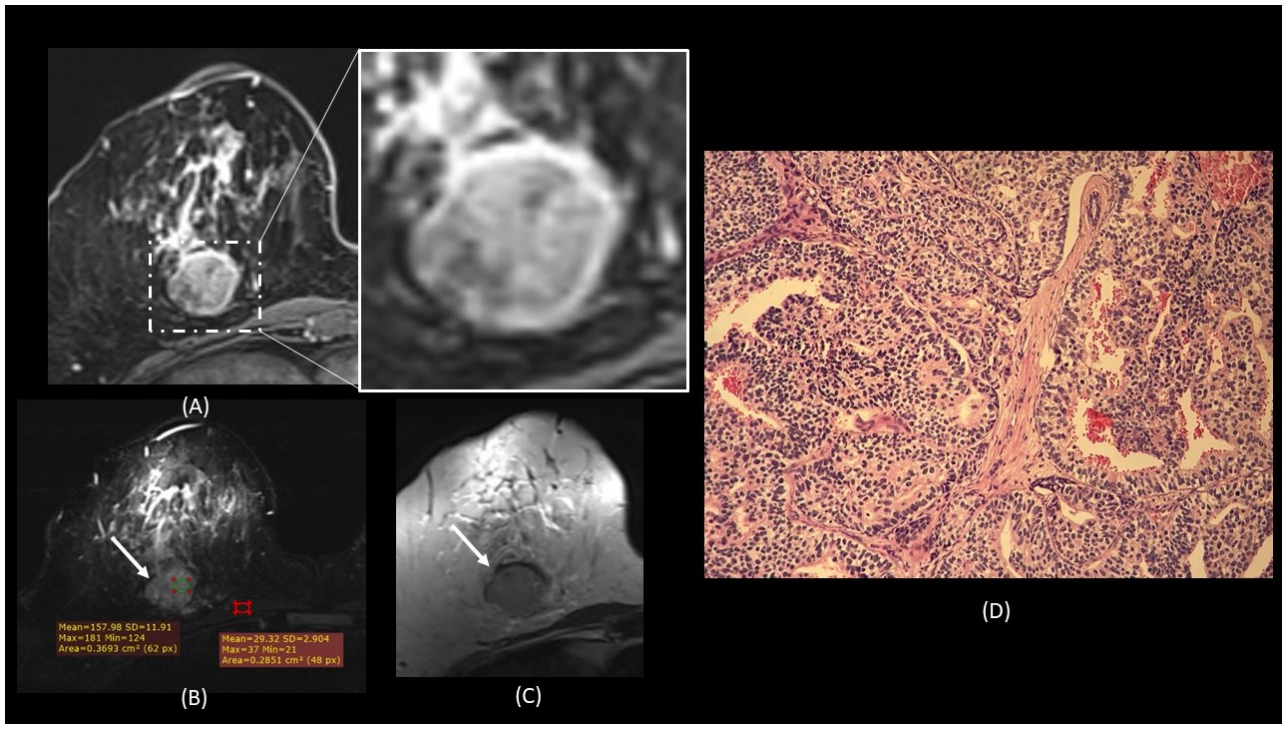
A 44-year-old lady with a palpable lump on the right breast. MRI T1- post contrast (A) showed a round, well-defined, rim-enhancing lesion at the right 6 o’clock position. (B) The STIR sequence is showing STIR signal intensity value of 158. (C) The T2-weighted signal intensity is showing iso-intensity. (D) Histopathology showing low tumour stroma, with the H+E, 10X: Low stromal group, photo shows mainly malignant breast cancer cells in nests and trabeculae architectures, background stromal is minimal.

**Fig 2:**
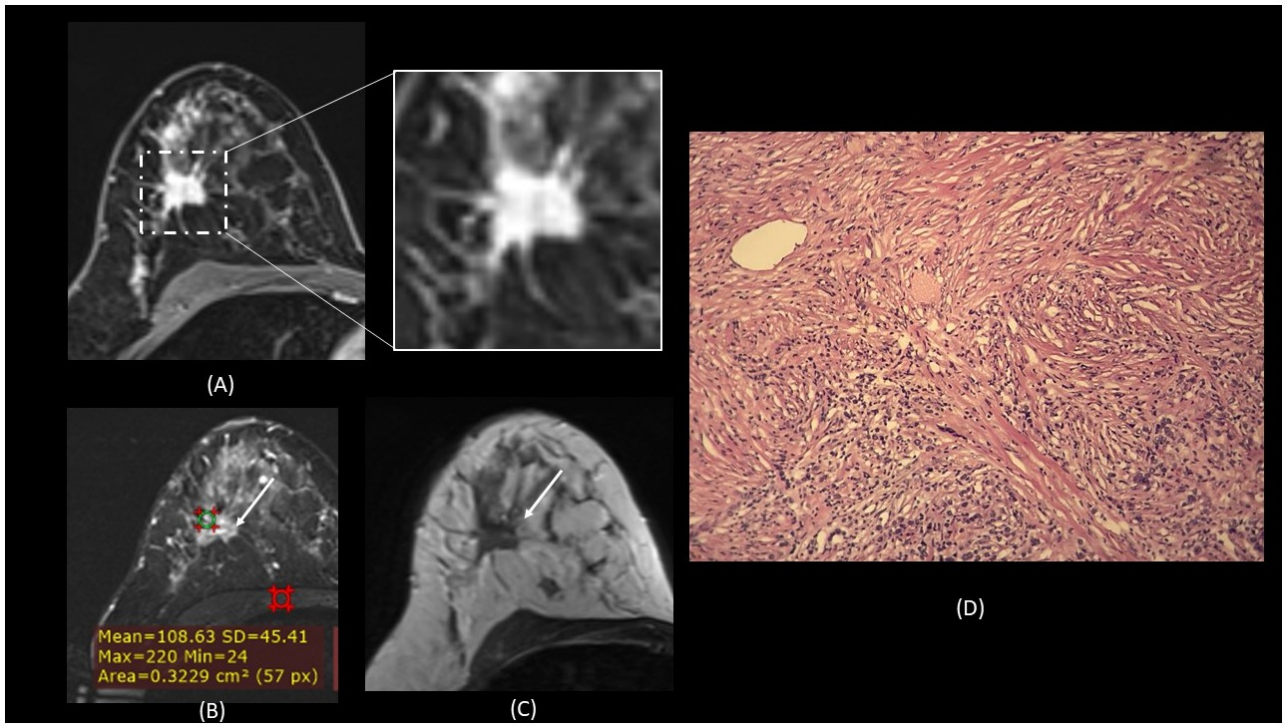
A 47 year-old lady with a palpable lump on the right breast. (A) MRI images in T1-post contrast showing an irregular, spiculated heterogeneously enhancing mass, with (B) STIR signal intensity of 108, and (C) is T2-weighted sequence demonstrating low-signal intensity. (D) Histopathology slide in H+E, 10X: Noted high stromal group, with large areas of hypocellular hyalinized stroma, and small clusters of malignant cells seen (lower right).

Cancer with high TSR were found to be associated with spiculated margin, whilst low TSR were well-circumscribed (Figs 1 and 2). Breast carcinoma with a prominent scirrhous reaction will reveal a spiculated margin and possess a strong fibrous area in the tumour’s central zone [14]. The fibrosis in invasive breast cancer with a scirrhous reaction and an infiltrative border was reported to be due to the fibroblastic proliferation caused by the production of basic fibroblast growth factor protein by the cancer cells. In contrast, cancers with a smooth border, the areas of coagulation necrosis appeared to be replaced by fibrosis, which suggested that the formation of a fibrotic focus in cancers with a smooth border were represented a repair phenomenon of necrosis [20]. Furthermore, cancers with spiculated margin on MRI has been associated with a relatively better prognosis than a well-circumscribed cancer [21]. This echoed our findings of spiculated masses were related to high TSR, hence the better prognosis.

Enhancement pattern in low TSR group is homogeneous and rim-enhancing, whilst in the high TSR group, the pattern was heterogeneous enhancement (Figs 1 and 2). Rim-enhancement was previously reported to be associated with triple negative cancer, which is associated with poor prognosis compared to the other subtypes [22,23]. Specifically, early rim enhancement was previously reported to be correlated with low peripheral to central fibrosis ratio and high peripheral to central micro vessel density ratio, whereas delayed rim enhancement was associated with peritumoral fibrosis [24].

Vascular endothelial growth factor (VEGF), which is an essential angiogenic peptide that induces neoangiogenesis and increased permeability was also previously correlated with early rim enhancement pattern. VEGF-A is the most researched and has significant relation to breast cancer intratumoural activity (29). The crosstalk between breast cancer and its microenvironment is one of the reasons for the tumour-associated stromal mediated mechanism of treatment resistance, for example in aromatase resistance may be caused by abnormal growth factor receptors expression [25]. This phenomenon is translated into imaging by the enhancement pattern observed on MRI.

We found an association between TSR and STIR signal intensity of the breast tumour, which is as previously reported by Yamaguchi et al [15]. High TSR group demonstrated lower STIR tumour signal intensity. Aside from breast cancer, other types of cancers, for example ovarian and desmoid tumour, had also showed low STIR signal intensity, which is likely due to the high fibrous component [26,27].

There was a significant difference between HER-2 receptor status and tumour stroma in our study, with high TSR cases associated with a positive HER-2 receptor. This is similar to previous reports by Gujam et al [28]. The HER receptor family is important in the regulation of normal breast development, however, overexpression of HER-2 is associated with development of breast cancer [29].

The limitation in our study includes the small sample size of a retrospective nature from a single institute. A larger sample size may help to give more statistically significant results. There is also no detailed pathological report aside from the biopsied sections. However, all cases included were discussed in multidisciplinary team meetings which involved the radiologist, breast surgeon and pathologist in charge to ensure the biopsied lesion corresponded to the MRI detected lesions.

### Conclusion

Breast cancer with high stroma had spiculated margin, lower STIR signal intensity, and heterogeneous pattern of enhancement in our study. Hence, in this preliminary works, certain MRI features showed a potential to predict TSR.

## Supporting information

S1 Table MRI Breast imaging parameters for 3.0T GE scanner and 3.0 siemens. (DOCX)

## References

[1] International Agency for Research on Cancer. GLOBOCAN 2020: Estimated cancer incidence, mortality and prevalence worldwide 2020 2020 [cited 2023 20 June 2023]. https://gco.iarc.fr/today/data/factsheets/populations/458-malaysia-fact-sheets.pdf.

[2] Mann RM, Cho N, Moy L. Breast MRI: State of the Art. Radiology. 2019;292(3):520–36. doi: 10.1148/radiol.2019182947 . 31361209

[3] Joseph C, Papadaki A, Althobiti M, Alsaleem M, Aleskandarany MA, Rakha EA. Breast cancer intratumour heterogeneity: current status and clinical implications. Histopathology. 2018;73(5):717–31. doi: 10.1111/his.13642 29722058

[4] de Kruijf EM, van Nes JG, van de Velde CJ, Putter H, Smit VT, Liefers GJ, et al. Tumor–stroma ratio in the primary tumor is a prognostic factor in early breast cancer patients, especially in triple-negative carcinoma patients. Breast cancer research and treatment. 2011;125(3):687–96. doi: 10.1007/s10549-010-0855-6 20361254

[5] Dekker T, Van De Velde C, Van Pelt G, Kroep J, Julien J, Smit V, et al. Prognostic significance of the tumor-stroma ratio: validation study in node-negative premenopausal breast cancer patients from the EORTC perioperative chemotherapy (POP) trial (10854). Breast cancer research and treatment. 2013;139(2):371–9. doi: 10.1007/s10549-013-2571-5 23709090

[6] TCGA CGAN. Comprehensive molecular portraits of human breast tumours. Nature. 2012;490(7418):61. doi: 10.1038/nature11412 23000897 PMC3465532

[7] Spratt DE, Evans MJ, Davis BJ, Doran MG, Lee MX, Shah N, et al. Androgen receptor upregulation mediates radioresistance after ionizing radiation. Cancer research. 2015;75(22):4688–96. doi: 10.1158/0008-5472.CAN-15-0892 26432404 PMC4651750

[8] Prat A, Perou CM. Deconstructing the molecular portraits of breast cancer. Molecular oncology. 2011;5(1):5–23. doi: 10.1016/j.molonc.2010.11.003 21147047 PMC5528267

[9] Haque R, Ahmed S, Schottinger J, Kwan M, Shi J, Chung J, et al. PS1-05: disparities in breast cancer survival by molecular subtype and race/ethnicity. Clinical medicine & research. 2012;10(3):145-.

[10] Onitilo AA, Engel JM, Greenlee RT, Mukesh BN. Breast cancer subtypes based on ER/PR and Her2 expression: comparison of clinicopathologic features and survival. Clinical medicine & research. 2009;7(1–2):4–13. doi: 10.3121/cmr.2009.825 19574486 PMC2705275

[11] Ahn S, Cho J, Sung J, Lee JE, Nam SJ, Kim K-M, et al. The prognostic significance of tumor-associated stroma in invasive breast carcinoma. Tumor Biology. 2012;33(5):1573–80. doi: 10.1007/s13277-012-0411-6 22581521

[12] Downey C, Simpkins S, White J, Holliday D, Jones J, Jordan L, et al. The prognostic significance of tumour–stroma ratio in oestrogen receptor-positive breast cancer. British journal of cancer. 2014;110(7):1744–7. doi: 10.1038/bjc.2014.69 24548861 PMC3974086

[13] Tozaki M, Fukuda K, Suzuki M. Dynamic high-spatial-resolution MR imaging of invasive ductal carcinoma: influence of histological scirrhous component on Mr descriptors. Magnetic Resonance in Medical Sciences. 2006;5(3):137–46. doi: 10.2463/mrms.5.137 17139139

[14] Matsubayashi RN, Imanishi M, Nakagawa S, Takahashi R, Akashi M, Momosaki S, et al. Breast ultrasound elastography and magnetic resonance imaging of fibrotic changes of breast disease: correlations between elastography findings and pathologic and short Tau inversion recovery imaging results, including the enhancement ratio and apparent diffusion coefficient. Journal of Computer Assisted Tomography. 2015;39(1):94–101. doi: 10.1097/RCT.0000000000000155 25299798

[15] Yamaguchi K, Hara Y, Kitano I, Hamamoto T, Kiyomatsu K, Yamasaki F, et al. Tumor-stromal ratio (TSR) of invasive breast cancer: correlation with multi-parametric breast MRI findings. The British journal of radiology. 2019;92(1097):20181032. doi: 10.1259/bjr.20181032 30835501 PMC6580921

[16] Roganovic D, Djilas D, Vujnovic S, Pavic D, Stojanov D. Breast MRI, digital mammography and breast tomosynthesis: comparison of three methods for early detection of breast cancer. Bosnian journal of basic medical sciences. 2015;15(4):64. doi: 10.17305/bjbms.2015.616 26614855 PMC4690445

[17] D’Orsi CJ, Sickles, Edward A, Mendelson, Ellen B, Morris Elizabeth A. ACR BI-RADS ATLAS Breast Imaging Reporting and Data System 2013. 5th edition ed. Reston, VA: American College of Radiology; 2013.

[18] Korkaya H, Liu S, Wicha MS. Breast cancer stem cells, cytokine networks, and the tumor microenvironment. The Journal of clinical investigation. 2011;121(10):3804–9. doi: 10.1172/JCI57099 21965337 PMC3223613

[19] Xu Q, Yuan J-P, Chen Y-Y, Zhang H-Y, Wang L-W, Xiong B. Prognostic significance of the tumor-stromal ratio in invasive breast cancer and a proposal of a new Ts-TNM staging system. Journal of oncology. 2020;2020. doi: 10.1155/2020/9050631 32377197 PMC7191412

[20] Hasebe T, Tsuda H, Hirohashi S, Shimosato Y, Tsubono Y, Yamamoto H, et al. Fibrotic focus in infiltrating ductal carcinoma of the breast: a significant histopathological prognostic parameter for predicting the long-term survival of the patients. Breast cancer research and treatment. 1998;49(3):195–208. doi: 10.1023/a:1006067513634 9776503

[21] Chang Y-W, Kwon KH, Choi DL, Lee DW, Lee MH, Lee HK, et al. Magnetic Resonance Imaging of Breast Cancer and Correlation with Prognostic Factors. Acta Radiologica. 2009;50(9):990–8. doi: 10.3109/02841850903225180 19863408

[22] Ab Mumin N, Hamid MTR, Wong JHD, Rahmat K, Ng KH. Magnetic Resonance Imaging Phenotypes of Breast Cancer Molecular Subtypes: A Systematic Review. Academic radiology. 2021. doi: 10.1016/j.acra.2021.07.017 34481705

[23] Navarro Vilar L, Alandete Germán SP, Medina García R, Blanc García E, Camarasa Lillo N, Vilar Samper J. MR Imaging Findings in Molecular Subtypes of Breast Cancer According to BIRADS System. The Breast Journal. 2017;23(4):421–8. doi: 10.1111/tbj.12756 28067435

[24] Tse GMK, Chaiwun B, Wong K-T, Yeung DK, Pang ALM, Tang APY, et al. Magnetic resonance imaging of breast lesions—a pathologic correlation. Breast Cancer Research and Treatment. 2007;103(1):1–10. doi: 10.1007/s10549-006-9352-3 17033923

[25] Criscitiello C, Esposito A, Curigliano G. Tumor–stroma crosstalk: targeting stroma in breast cancer. Current Opinion in Oncology. 2014;26(6):551–5. doi: 10.1097/CCO.0000000000000122 25279962

[26] Imaoka I, Wada A, Kaji Y, Hayashi T, Hayashi M, Matsuo M, et al. Developing an MR Imaging Strategy for Diagnosis of Ovarian Masses. RadioGraphics. 2006;26(5):1431–48. doi: 10.1148/rg.265045206 . 16973774

[27] Braschi-Amirfarzan M, Keraliya AR, Krajewski KM, Tirumani SH, Shinagare AB, Hornick JL, et al. Role of Imaging in Management of Desmoid-type Fibromatosis: A Primer for Radiologists. RadioGraphics. 2016;36(3):767–82. doi: 10.1148/rg.2016150153 . 27163593

[28] Gujam FJA, Edwards J, Mohammed ZMA, Going JJ, McMillan DC. The relationship between the tumour stroma percentage, clinicopathological characteristics and outcome in patients with operable ductal breast cancer. British Journal of Cancer. 2014;111(1):157–65. doi: 10.1038/bjc.2014.279 24874480 PMC4090742

[29] Yarden Y. Biology of HER2 and Its Importance in Breast Cancer. Oncology. 2001;61(suppl 2)(Suppl. 2):1–13. doi: 10.1159/000055396 11694782

